# Is the grass always greener? Land surface phenology reveals differences in peak and season‐long vegetation productivity responses to climate and management

**DOI:** 10.1002/ece3.7904

**Published:** 2021-07-22

**Authors:** David J. A. Wood, Scott Powell, Paul C. Stoy, Lindsey L. Thurman, Erik A. Beever

**Affiliations:** ^1^ U.S. Geological Survey Northern Rocky Mountain Science Center Bozeman Montana USA; ^2^ Department of Land Resources and Environmental Sciences Montana State University Bozeman Montana USA; ^3^ Department of Biological Systems Engineering University of Wisconsin–Madison Madison Wisconsin USA; ^4^ U.S. Geological Survey Northwest Climate Adaptation Science Center Corvallis Oregon USA; ^5^ Department of Ecology Montana State University Bozeman Montana USA

**Keywords:** disturbance, grasslands, productivity, remote sensing, shrub‐steppe, trade‐offs

## Abstract

Vegetation phenology—the seasonal timing and duration of vegetative phases—is controlled by spatiotemporally variable contributions of climatic and environmental factors plus additional potential influence from human management. We used land surface phenology derived from the Advanced Very High Resolution Radiometer and climate data to examine variability in vegetation productivity and phenological dates from 1989 to 2014 in the U.S. Northwestern Plains, a region with notable spatial heterogeneity in climate, vegetation, and land use. We first analyzed interannual trends in six phenological measures as a baseline. We then demonstrated how including annual‐resolution predictors can provide more nuanced insights into measures of phenology between plant communities and across the ecoregion. Across the study area, higher annual precipitation increased both peak and season‐long productivity. In contrast, higher mean annual temperatures tended to increase peak productivity but for the majority of the study area decreased season‐long productivity. Annual precipitation and temperature had strong explanatory power for productivity‐related phenology measures but predicted date‐based measures poorly. We found that relationships between climate and phenology varied across the region and among plant communities and that factors such as recovery from disturbance and anthropogenic management also contributed in certain regions. In sum, phenological measures did not respond ubiquitously nor covary in their responses. Nonclimatic dynamics can decouple phenology from climate; therefore, analyses including only interannual trends should not assume climate alone drives patterns. For example, models of areas exhibiting greening or browning should account for climate, anthropogenic influence, and natural disturbances. Investigating multiple aspects of phenology to describe growing‐season dynamics provides a richer understanding of spatiotemporal patterns that can be used for predicting ecosystem responses to future climates and land‐use change. Such understanding allows for clearer interpretation of results for conservation, wildlife, and land management.

## Graphical abstract

We compared interannual trends in six satellite‐derived phenological measures across seven vegetation community types to models incorporating climate in the U.S. Northwestern Plains. We found more nuanced insights by including climate over just intrannual phenological trends, that relationships between climate and phenology varied across the region, and responses of phenological measures did not covary with drivers. In sum, analyses should not assume climate alone drives patterns and areas may exhibit different patterns of greening or browning when using peak versus season‐long productivity.

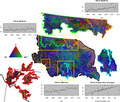

## 1 INTRODUCTION

Ecological information across space and time can enhance decision‐making by allowing researchers and practitioners to address problems within appropriate geographies, derive landscape‐scale indicators of change, and prioritize in situ monitoring efforts (Dale et al., 2000; Lausch et al., 2018; Marvin et al., 2016). For example, adequate understanding of the timing, magnitude, and duration of seasonal events (phenology) of an ecological community can inform management and restoration efforts and help managers estimate how sensitive a community may be to ongoing changes in climate. Conversely, not accounting for spatiotemporal patterns in phenology can lead to mismanagement, including food‐security risks (e.g., Bezerra et al., 2019; Sadras & Monzon, 2006; Stevenson et al., 2015), ecological disruption caused by phenological mismatches (e.g., Rehnus et al., 2020; Renner & Zohner, 2018), and sampling biases (Gibson et al., 2016; Smith et al., 2017). Vegetation phenology interacts with climate and has cascading impacts to ecosystem processes such as nutrient cycling and the maintenance of ecosystem services (Beard et al., 2019; Cleland et al., 2007; Morisette et al., 2009). Furthermore, wildlife species within a system may respond to vegetation phenology and productivity with potential cascading effects on seasonal timings, fecundity, interspecific interactions, and behavior (Donnelly et al., 2018; Rehnus et al., 2020; Stoner et al., 2020).

Many aspects of phenology can be measured by satellite‐based remote sensing, termed “land surface phenology,” a combined measurement of variation across multiple plant species and individuals as well as background surfaces such as soils (Hanes et al., 2014). Studies of land surface phenology have identified numerous changes across the globe in the seasonal cycle of vegetation growth due to climate, including changes to start of spring, end of season, and season‐long productivity (Chen et al., 2020; Morisette et al., 2009). Greening, measured through either increased growing‐season production or higher peak productivity, is evident over considerable portions of the Earth's terrestrial surface (Piao et al., 2019; Zhu et al., 2016). However, parts of the terrestrial surface have experienced no change or are browning—a decrease in growing‐season production and/or lower peak productivity (de Jong et al., 2011; Meng et al., 2020; Zhu et al., 2016). Trends in greening, browning, and other aspects of phenological change often challenge the predictions of land surface models, suggesting that our understanding of these processes must be improved.

Phenological measurements, be they dates or magnitudes, are not always strictly increasing or decreasing; many factors play into their variability and patterns (de Jong et al., 2011; Li et al., 2019; Piao et al., 2019). Although analyses of an individual phenological measure through time (interannual trend) provide important information, the amount of the land surface that exhibits statistically significant trends in these phenological measurements reflects how constraining climate factors are changing through time and how ecosystems respond to these changes. Interannaul trends may depend heavily on the selected starting and ending years of the time series, which may mask more subtle changes in the progression of vegetation greenness in an area (e.g., Yang et al., 2021; Yuan et al., 2019). Including climatic drivers in models of phenology helps elucidate the mechanisms behind observed changes across years. In addition, there are important differences in the implications of climate change for peak productivity versus season‐long productivity. Unfortunately, only peak or season‐long productivity is typically investigated, which can obscure comparisons of the mechanisms underlying different aspects of phenological changes (Gao et al., 2020). Precipitation and temperature have been identified as important drivers of seasonal variation in productivity; however, these relationships can vary across ecosystems and vegetation types (Fu et al., 2017; Maurer et al., 2020; Reed et al., 2019; Yang et al., 2021). Furthermore, other natural and anthropogenic factors may confound this spatial dynamic, such as disturbance, land‐use and land‐cover change (including invasive species), CO_2_ enrichment, and changes to water use and soil water‐holding capacity (Nemani et al., 2003; Piao et al., 2019; Zhang et al., 2019).

**FIGURE 1:**
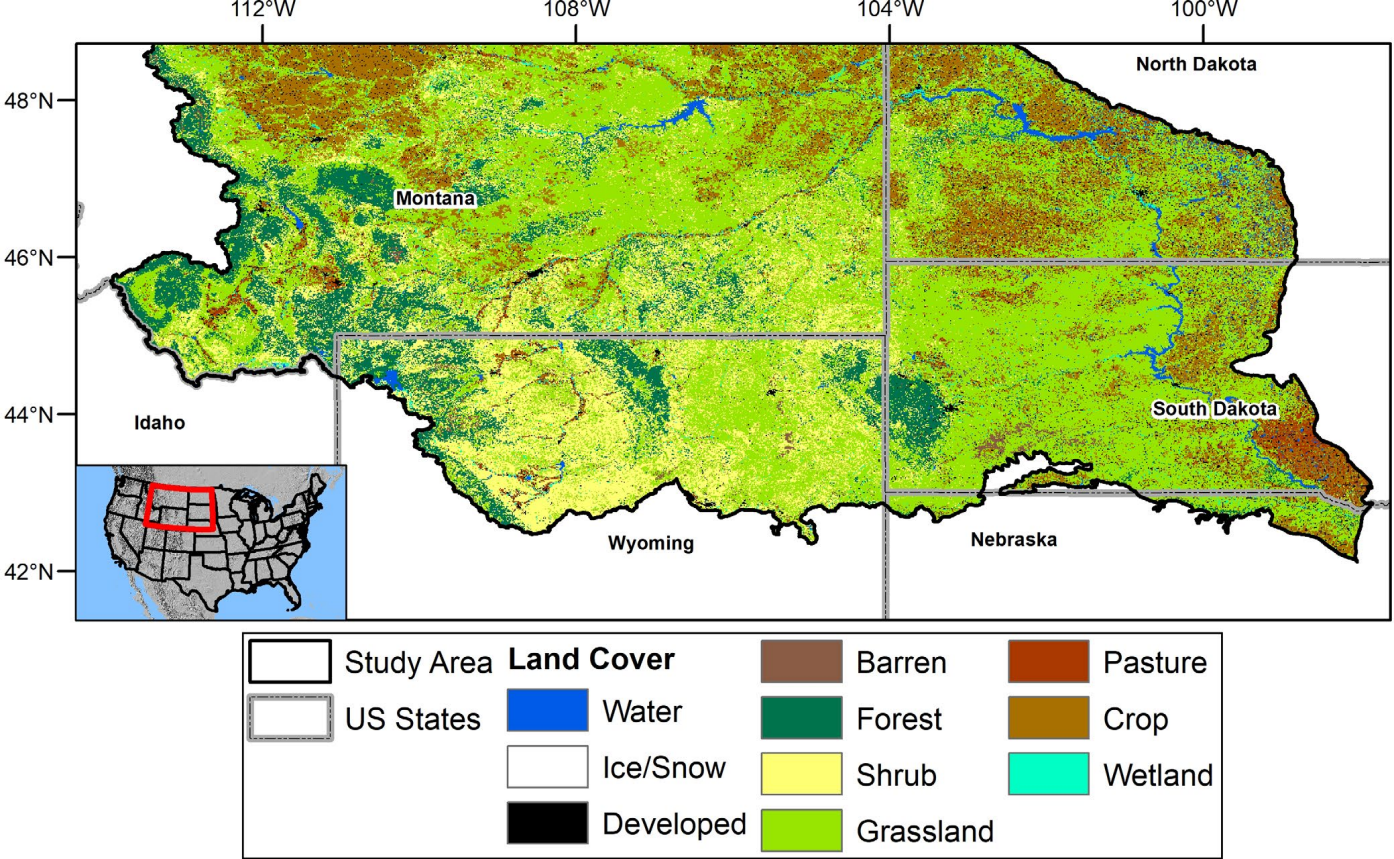
The Northwestern Plains study area, which comprises the Northwestern Great and Glaciated Plains Level 3 Ecoregions (Omernik, 1987), plus areas containing semi‐arid vegetation in the upper Missouri River basin. These additions include portions of the Middle Rocky Mountains and Wyoming Basins Ecoregions. Land cover data from the Multi‐Resolution Land Characteristics Consortium (NLCD 2016, available at www.mrlc.gov)

**FIGURE A1:**
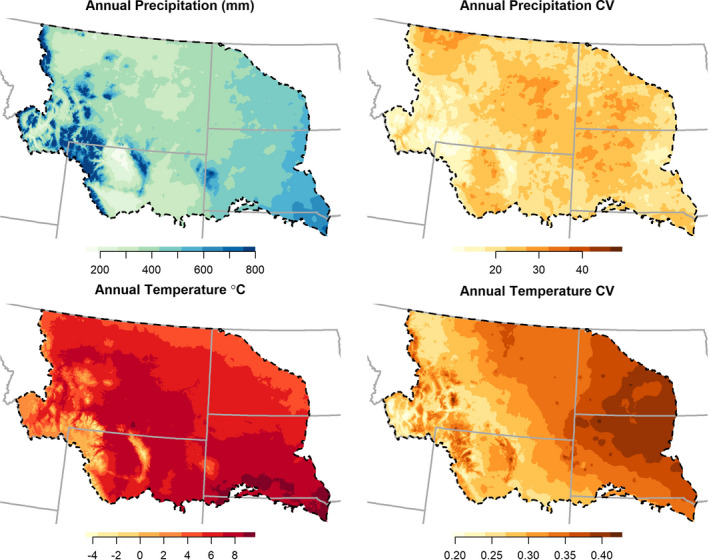
Mean annual temperature and precipitation, and their coefficient of variation (CV) in the Northwestern U.S. Great Plains from 1989 to 2014, coinciding with the AVHRR satellite‐derived phenological record. Precipitation values limited to a value of 800 mm/year for display purposes (i.e., the dark blue is 800+ mm), ranging up to 2,500 mm in higher elevation areas in the Rocky Mountains. In order to use a ratio scale for the coefficient of variation calculation on temperature values, yearly data were first converted to kelvin temperatures (℃ + 273.15). The study area is depicted with a dashed black line and U.S. states with gray solid lines

The U.S. Northwestern Plains (NWP; Figure 1) provide a meaningful geography to expand from prior studies of phenological change due to large expanses of intact native vegetation (Auch et al., 2011) interspersed by more intensively managed agricultural areas. Climatic drivers of phenology vary across gradients in the NWP, which exhibits strong west‐to‐east increases in precipitation and south‐to‐north decreases in temperature (Figure A1; Epstein et al., 1996). Interannual variability in precipitation is high in the NWP, leading to variable productivity (Petrie et al., 2016); however, productivity of higher elevation areas, which are typically forested, has varied less over time and has responded more strongly to temperature than precipitation (Fu et al., 2017; Potter, 2020). Collectively, these factors lead to variability in spatiotemporal patterns of phenology across the NWP. Here, interannual trends of increasing productivity outweigh browning trends; about 20% of the NWP has exhibited a significant increase in peak productivity (as measured by maximum annual NDVI) over the past few decades (Brookshire et al., 2020), and further increases are anticipated (Hufkens et al., 2016). Additionally, growing‐season length has also increased, primarily due to later end‐of‐season dates rather than earlier start‐of‐season dates (Ren et al., 2020). How these different measurements of phenology combine to determine changes in vegetation seasonality and productivity across different ecosystems in the NWP remains less clear.

Consequently, we aimed to examine the spatiotemporal variability in dates and magnitudes of phenological events in the NWP and how prevailing climatic conditions may influence and/or govern this variability. Ecological models, in our case phenological ones, can be hierarchical, complex, and challenging to parameterize (Morisette et al., 2009; Newman et al., 2019). Therefore, to explore patterns at various scales, for example, temporal, regional, and community level, we analyzed spatiotemporal patterns in six phenological metrics across seven plant communities and an entire ecoregion. Specifically, our objectives were to first evaluate interannual trends and then compare those findings to a model incorporating annual precipitation and temperature across space and vegetation community types of the NWP (Figure 1). We then used these findings to examine the predictive ability of annual weather variables for phenological measures and evaluate their role in patterns, such as variation of greening versus browning between different measures of productivity.

## 2 MATERIALS AND METHODS

### 2.1 Study area

We defined the NWP study area using a two‐part process. First, we selected the Northwestern Great and Glaciated Plains ecoregions (https://www.epa.gov/eco‐research/ecoregions; Omernik, 1987) and then included semi‐arid areas within the upper Missouri River basin, the transitional areas from the Great Basin (parts of the Middle Rocky Mountains) and Wyoming Basins (Bighorn Basin part of this ecoregion, Figure 1). We chose this boundary to include similar vegetation types of semi‐arid grasslands and shrublands with intermixed agricultural and forested areas and to cover a wide range of climatic regimes. Vegetation communities range from barren and shrubland systems in the west to mixed‐grass and tall‐grass prairie to the east. Forested areas are found in higher elevations, such as the western edge of the NWP, in mountain islands like the Black Hills, and adjacent to rivers. Areas converted to agriculture primarily occur in the northern and eastern portions of the study region and in irrigated areas along waterways. The NWP receives from 33 to 56 cm (13 to 22 in) of precipitation per year and mean annual temperature ranges from 3.8 to 10.5℃ (39 to 51℉), although precipitation can exceed 160 cm (63 in) per year, and annual mean temperatures average below 0℃ (32℉) at higher elevations (Figure A1; USDA, 2006).

### 2.2 Data sources and processing

We obtained phenology data derived from the Advanced Very High Resolution Radiometer (AVHRR) satellite processed by the USGS for the years 1989–2014 (USGS EROS, 2015). These data utilized composites of daily, 1 km pixel values to calculate the normalized difference vegetation index (NDVI), from which phenological measures were derived (Eidenshink, 2006; Reed et al., 1994). The general approach applied by the USGS Remote Sensing Phenology Center for these data is to create smoothed NDVI composites (Eidenshink, 2006) using a weighted least squares approach (Swets, 1999) and employ a delayed moving‐average algorithm to identify dates of phenological events and their magnitudes (Reed et al., 1994). Due to degradation in the orbit at the end of life for different platforms carrying the AVHRR, solar zenith angles (SZAs) have changed through time and are associated with trends in phenological measures, degrading the temporal consistency (Ji & Brown, 2017) and precluding analyses beyond 2014. Although much of the study area had limited area with significant correlations between SZA and phenological measures, we chose to remove problematic years (1992, 1993, 1994, 1999, and 2002) to minimize issues from orbital degradation (Ji & Brown, 2017). Our comparison of results including versus excluding the five problematic years found limited large‐scale effects on the results.

**FIGURE 2:**
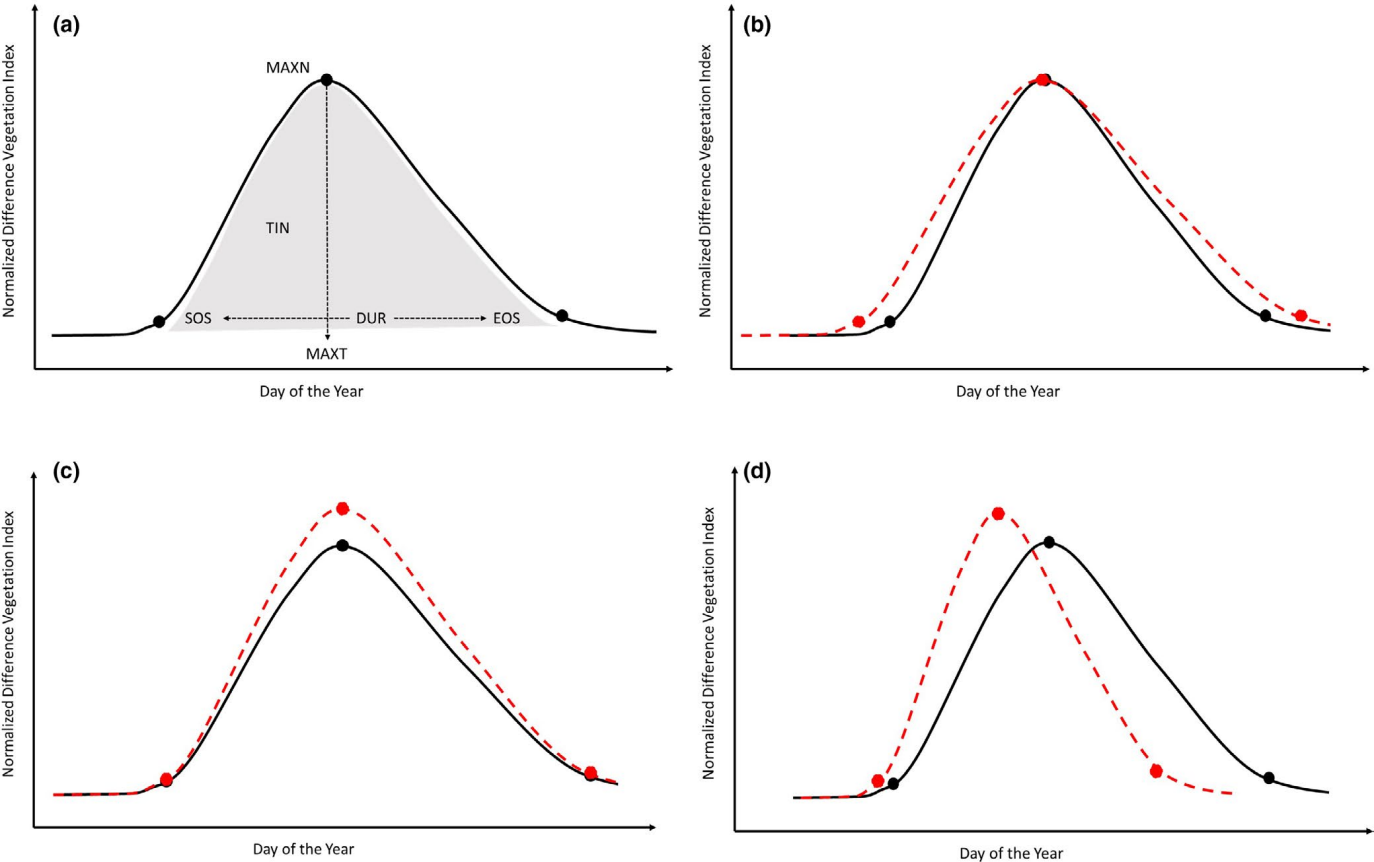
Visualization of phenological measures from the smoothed normalized difference vegetation index (NDVI) used in this study (a). These include the Julian day for start of season (SOS), end of season (EOS), and date of maximum NDVI (MAXT), and growing‐season characteristics of season duration (DUR = EOS − SOS), value of maximum NDVI (MAXN), and time‐integrated NDVI (TIN, shaded gray area). Potential changes (red dashed lines) to the growing‐season curve include extending the growing season through earlier SOS and later EOS with a similar MAXT but higher TIN (b), increasing the MAXN but with similar SOS and EOS leading to a higher TIN (c), and shifting to an earlier SOS and MAXT, with a higher MAXN, earlier EOS, and overall lower TIN (d). Other combination changes to SOS, EOS, MAXT, and MAXN are possible with cascading effects on TIN

We used six phenological measures (Figure 2) similar to those used in other studies of semi‐arid systems (Maurer et al., 2020; Ren et al., 2020; Yang et al., 1998). These included four date‐related measures (start, end, and length of season, and day of peak productivity (NDVI)) and two measures related to productivity and the shape of the phenological curve (maximum NDVI and time‐integrated NDVI). While maximum NDVI (hereafter peak productivity) is a surrogate for vegetation productivity at a single point in time, the peak of the growing season, time‐integrated NDVI (hereafter season‐long productivity) is a surrogate for vegetative productivity across the entire growing season.

We acquired climate data from the Parameter‐elevation Relationships on Independent Slopes Model (PRISM) version AN81d at 30‐arcseconds (~800 m, for our study domain) spatial resolution (Daly et al., 2008) for each calendar year of our phenology dataset (1989–2014). We chose annual values (Figure A1) to provide a parsimonious overview of the complex mix of phenological measures and vegetative communities. In effect, annual measures are a relative index of the average climate conditions that each “pixel” experiences over the study period. We recognize the decreased fit and sensitivity that may occur by combining seasons, in addition to a reduction in identifying the mechanistic understanding of seasonal factors influencing phenology. By reducing the complexities, this approach allowed us to assess whether relatively simple models provide meaningful predictions that can connect with annual climatic variability.

**FIGURE A2:**
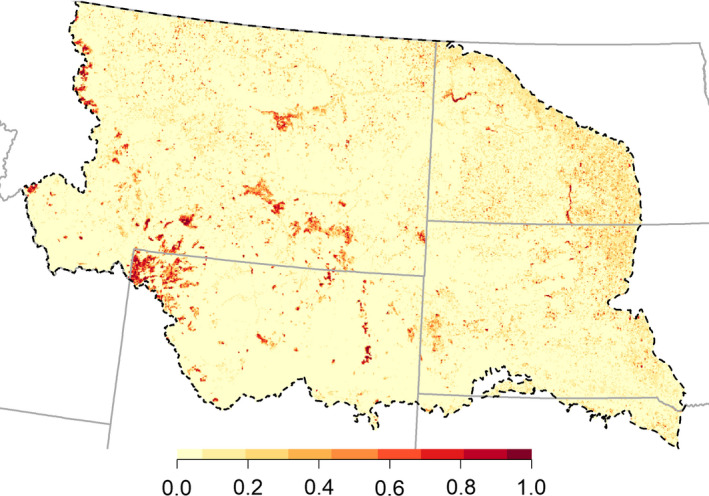
Proportion of AVHRR pixels (1 km resolution) experiencing land‐cover type change between 2001 and 2016. Land‐cover changes derived from the Landsat‐based (30 m resolution) NLCD Change Index record. The study area is depicted with a dashed black line and U.S. states with gray solid lines

For land cover, we utilized the 2016 National Land Cover Database (Figure 1), a Landsat‐derived classification of cover across the continental United States that includes data every 2–3 years between 2001 and 2016 (Wickham et al., 2014, Yang et al., 2018). There are sixteen classes, including developed, agricultural, and natural land‐cover classes. We aggregated these 30‐m pixels to assign the primary (modal) land‐cover class within each 1‐km AVHRR pixel for 2001, 2004, 2006, 2008, 2011, and 2013. We then identified AVHRR pixels where the modal land‐cover class remained the same for every year and used those in analyses by land‐cover type. Values for land surface phenology measures over heterogeneous areas can be driven by a subset of the area (e.g., a subdominant land‐cover class) (Zhang et al., 2017). Therefore, we also used the change index NLCD layer to calculate the per AVHRR pixel (1 × 1 km resolution) percentage of Landsat pixels (30 × 30 m resolution) that changed between 2001 and 2016. This index was used to measure any subdominant land‐cover changes in the study area (Figure A2) to assess whether such changes may be influencing trends. Our analysis considered the land‐cover classes of barren (i.e., vegetation generally minimal or constituting a minority of the pixel, e.g., South Dakota badlands), deciduous forest, evergreen forest, shrubland, grassland, agriculture (a combination of pasture, intensively managed under an annual or perennial cycle for feed, grazing, crops, and/or woody agriculture), and wetlands (combined woody and herbaceous wetlands) (Table 1).

**TABLE 1:** Pixel numbers for analyzed community types and proportion class of pixels with minor type changes between 2001 and 2016

Land‐cover type	Area (km^2^)	Proportion of study area	Proportion of type area for each change per pixel percentage category				
			0–20%	20–40%	40–60%	60–80%	80–100%
Barren	2,368	0.4%	96.2%	2.5%	1.0%	0.2%	0.0%
Deciduous forest	469	0.1%	97.4%	2.6%	0.0%	0.0%	0.0%
Evergreen forest	54,783	8.5%	94.1%	4.3%	1.2%	0.3%	0.2%
Shrub	146,987	22.8%	98.2%	1.4%	0.3%	0.1%	0.0%
Grassland	309,241	48.0%	95.3%	3.7%	0.8%	0.1%	0.0%
Pasture/crop	119,221	18.5%	94.1%	5.2%	0.5%	0.1%	0.0%
Wetland	3,937	0.6%	91.6%	7.0%	1.3%	0.1%	0.0%
Other	41,911	1.4%	41.4%	24.2%	16.1%	11.7%	6.5%
Total	679,024	100.0%	92.3%	4.8%	1.6%	0.8%	0.4%

Note

Area calculated from the AVHRR pixels where the modal land‐cover type is unchanged between 2001 and 2013 based on a Landsat‐based classification including NLCD 2001, 2004, 2006, 2008, 2011, and 2013. The “Other” land‐cover type includes AVHRR pixels where the modal land‐cover type changed over the time period. Change classes based on the NLCD 2016 Land Cover Change index, as the proportion of 30‐m pixels coded under any change category within each AVHRR pixel.

### 2.3 Data analysis

We conducted two sets of analyses to demonstrate how the spatial variation in phenology measures and observations, such as greening, can be enriched with the inclusion of climate variables that are likely drivers of potential trends. First, we modeled interannual trends for each of our six phenology measures (Figure 2). For each AVHRR pixel, we calculated the Sen's slope to account for outliers, skewed data, and heteroscedasticity and used the Mann–Kendall test to assess significance of the interannual trend. For each phenology measure, we removed any record with more than three total missing values and then interpolated any gaps of one value. Any remaining records with missing data (gaps of two or more years) were removed. As Sen's slope requires a continuous record, we included all AVHRR years in this analysis, but removed years with orbital degradation from the Mann–Kendall test of the significance of the trend. We used the land‐cover data to examine the distribution of Sen's slopes between cover classes.

Second, to identify associations of climate factors in addition to time‐based trends in phenological measures, we fit linear models including the explanatory variables of annual mean temperature, annual precipitation, and time. Our model for each AVHRR pixel was:

(1) $$Y_{i}=B_{0}+B_{1}Year_{i}+B_{2}P_{i}+B_{3}T_{i}+\varepsilon_{i}$$

where *Y_i_* is the *i*th observation of the phenological measure (dependent variable), *B*_0_ is the intercept, *B*_1…_ *B_k_* are the slope coefficients for independent variables, *Year_i_* is the *i*th year of the observation, *P_i_* is the total annual precipitation in the *i*th year, *T_i_* is the annual mean temperature in the *i*th year, and *ε_i_* is the error term for the *i*th observation. This allows us to expand beyond simple trend analyses to disentangle associations of time, precipitation, and temperature. We included time (*Year*) as a variable to represent temporally dependent linear effects that are not accounted for by temperature and precipitation measures, such as changes to land management or use, disturbance recovery, other climatic factors including increasing atmospheric CO_2_ concentrations, or sensor effects (e.g., Auch et al., 2011; Brookshire et al., 2020; Nemani et al., 2003; Stoy et al., 2018; Zhu et al., 2016). To display the relative contributions of each predictor across space, we calculated their partial correlation. Then, we took the absolute value of these and normalized values to cover the range of 0–255 so each independent variable could be used as an input band into a RGB image. This approach is used to visualize the within‐study‐area contribution of each covariate for this analysis and identify the dominant associations for each phenological measure. All statistical analyses were performed in R Studio (Version 1.1.463) using R 4.0.3 (R Core Team, 2019) and the R packages *ppcor* (Kim, 2015), *raster* (Hijmans, 2019), *sp* (Bivand et al., 2008; Pebesma & Bivand, 2005), *rgdal* (Bivand et al., 2019), *trend* (Pohlert, 2020), and *zoo* (Zeileis & Grothendieck, 2005) for analyses, and *RColorBrewer* (Neuwirth, 2014) and *Plot3D* (Soetaert, 2017) for display.

## 3 RESULTS

### 3.1 Phenology trends

**FIGURE 3:**
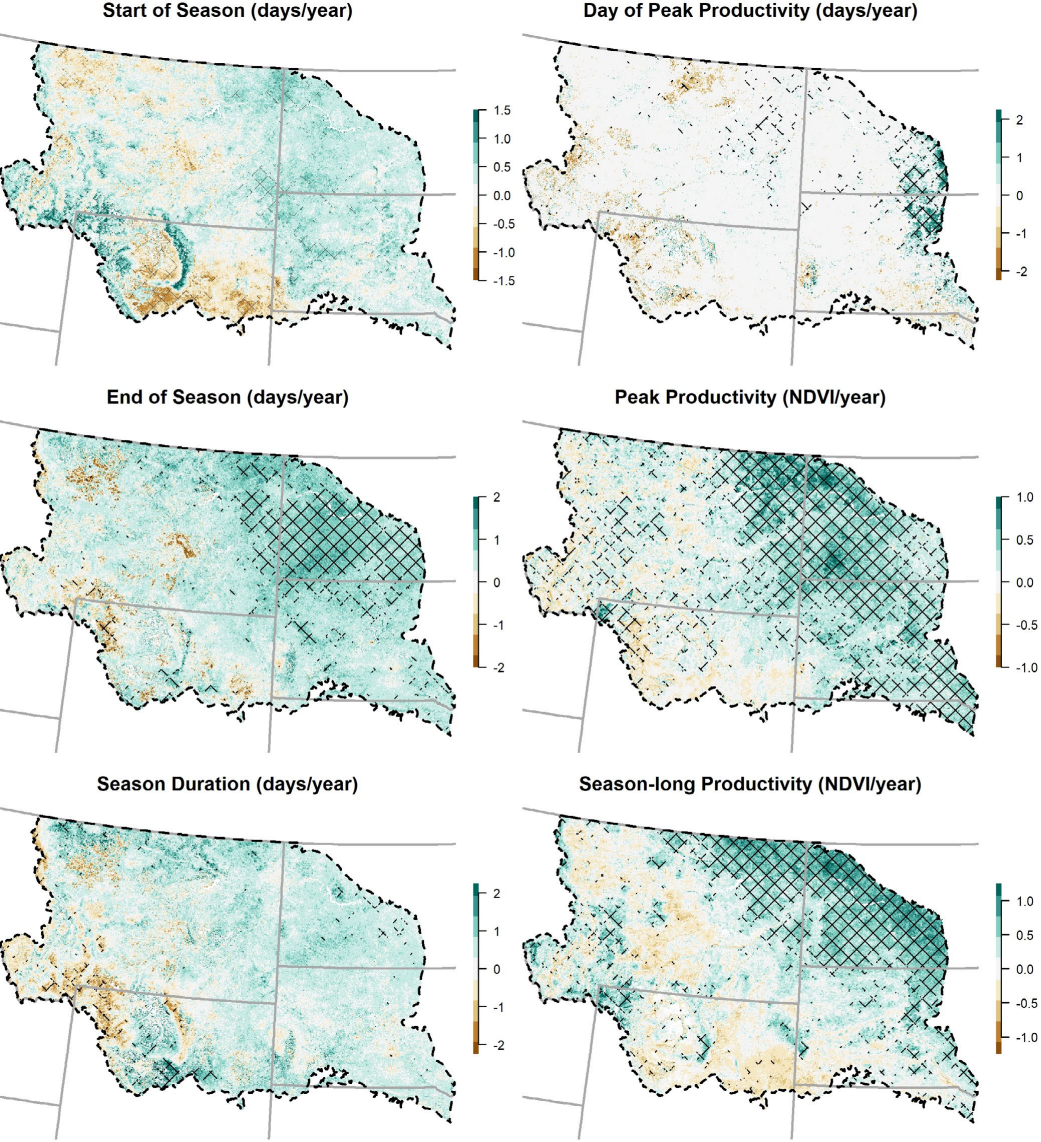
Interannual phenological trends (with units in the title of each plot) for 1989–2014 based on the AVHRR satellite record (values from Sen's slope). The study area is depicted with a dashed black line and U.S. states with gray solid lines. Hatching indicates areas where Mann–Kendall tests for trend were significant (*p* < .05)

Notable patterns emerged from the interannual trends of phenological measures (Figure 3; Table A1). For example, the majority of trends for the start and end of season increased, suggesting both are occurring later, but less than 15% of AVHRR pixels exhibited significant trends. The majority of interannual trends toward earlier start‐of‐season days were found in the southern portion of the study domain, whereas the northeastern portion of the study area was characterized by later end of season and some later days of peak productivity (Figure 3). Season‐long productivity had significant trends for 23% of AVHRR pixels in the study area, the majority of which (84%) increased. Similarly, 29% of pixels had significant trends for peak productivity of which 95% increased. The larger increases in season‐long productivity and peak productivity were in the northeastern NWP, whereas the majority of decreases occurred in the southwestern portion of the study area (Figure 3).

**FIGURE A3:**
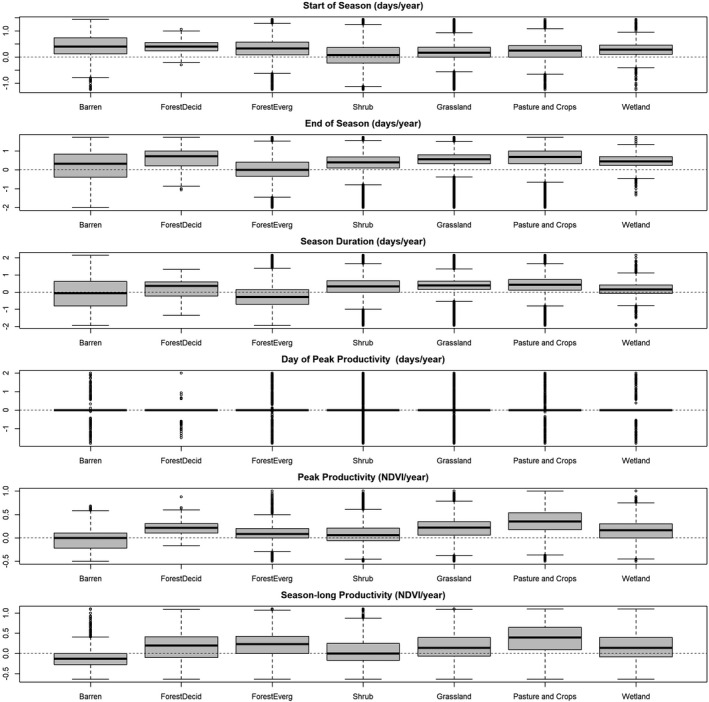
Boxplots of Sen's slopes for six phenology measures by vegetation type (ForestDecid = Deciduous Forest, ForestEverg = Evergreen Forest, see text for description of each land‐cover class) for 1989–2014 based on the AVHRR satellite record. Boxplots represent the distribution of slopes for each phenological measure, with the dashed line representing no change (zero slope)

**FIGURE A4:**
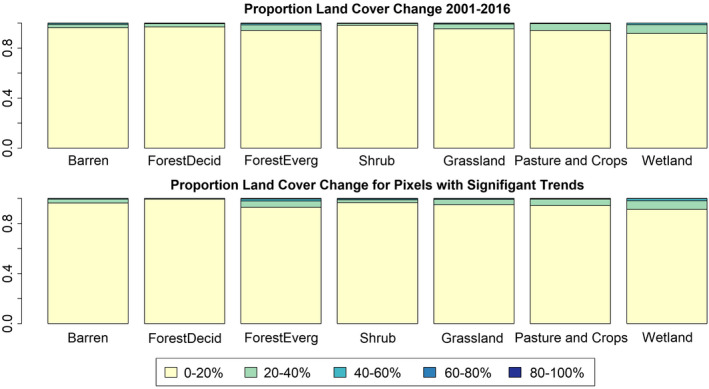
Stacked proportional bar plot comparison of study area wide change of land‐cover types (ForestDecid = Deciduous Forest, ForestEverg = Evergreen Forest, see text for description of each land‐cover class) within AVHRR pixels to those with significant interannual trend of peak productivity (MAXN). Land cover derived from the Landsat‐based (30 m) NLCD Change Index record. MAXN used as the example comparison as it has the largest area of significant interannual trend and overlap with the majority of other significant interannual trends for the other five phenological measures studied

Significant trends in phenological measures varied by plant community type and responses were not synchronous, and trends are likely only associated with major land‐cover changes in a small number of cases (Tables 1 and A1; Figures A3 and A4). Some notable exceptions between land‐cover classes for phenological measures include shrub communities having a balanced split between trends of earlier (48%) and later (52%) spring start compared to other communities wherein the preponderance of significant AVHRR pixels moved toward later spring onset (>70%). Evergreen and deciduous forests and barren areas tended toward an earlier end of season, while other communities tended toward later end‐of‐season dates. Significant trends in peak productivity (95%) and season‐long productivity (96%) largely increased; however, barren communities and to some extent evergreen forests differed from other communities with less dominant splits between increasing and decreasing peak and season‐long productivity. Between 2001 and 2016, 94% of the study area (as measured in changes to Landsat pixels) remained in the same land‐cover class (Figure A2) and the vast majority (92%) of the AVHRR pixels in the study area had limited, less than 20%, subpixel change (Table 1). AVHRR pixels with significant trends in phenological measures were not more likely to have increased subpixel cover class changes (Figure A4).

### 3.2 Associations of climate and time to phenology measures

**FIGURE 4:**
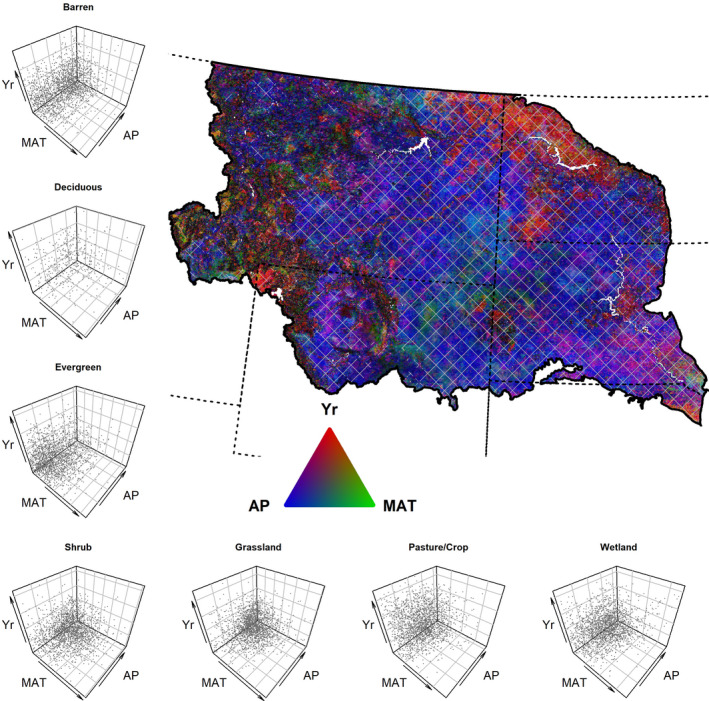
The combination of partial correlation coefficients for different drivers on maximum vegetation productivity. Scatter plots (left column and bottom row) and RGB image (upper right) illustrate the partial correlations of annual precipitation (AP), mean annual temperature (MAT), and time (Yr) on productivity as measured by yearly AVHRR maximum NDVI from 1989 to 2014. The thick solid line represents the study area boundary, and the dashed lines are U.S. state boundaries. The scatter plots identify the absolute values of partial correlation coefficients from 2000 randomly selected pixels from the study for each vegetation community. In the map, color represents the relative association (absolute values) of the correlation of each factor. The brightness of pixels is relative to the combined partial correlation where darker colors have smaller and brighter colors have larger summed partial correlations. Hatching indicates areas where at least one partial correlation coefficient of the three variables considered was significant (*p* < .05)

**FIGURE 5:**
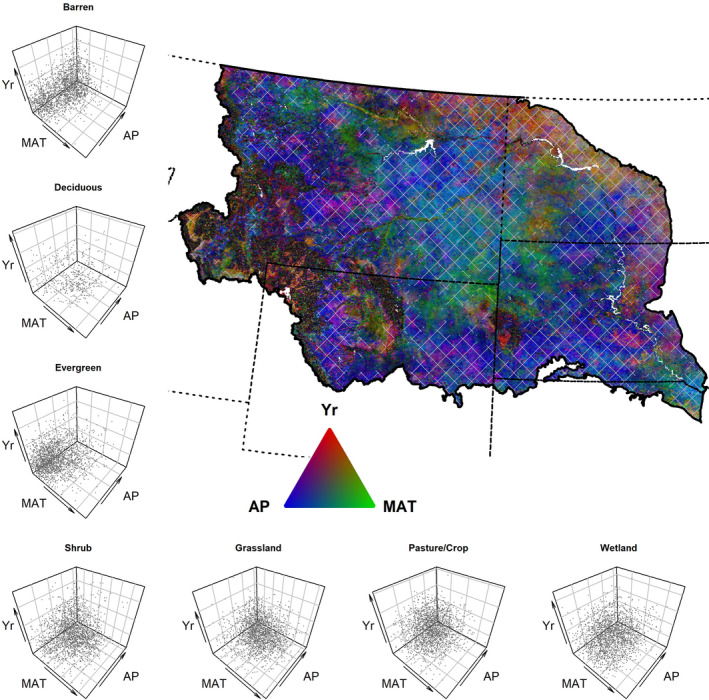
The combination of partial correlation coefficients for different drivers on season‐long vegetation productivity. Scatter plots (left column and bottom row) and RGB image (upper right) illustrate the partial correlations of annual precipitation (AP), mean annual temperature (MAT), and time (Yr) on productivity as measured by AVHRR time‐integrated NDVI from 1989 to 2014. The thick solid line represents the study area boundary, and the dashed lines are U.S. state boundaries. The scatter plots identify the absolute values of partial correlations from 2000 randomly selected pixels from the study for each vegetation community. In the map, color represents the relative association (absolute values) of the correlation of each factor. The brightness of pixels is relative to the combined partial correlations where darker colors have smaller and brighter colors have larger summed partial correlations. Hatching indicates areas where at least one partial correlation coefficient of the three variables considered was significant (*p* < .05)

**FIGURE A5:**
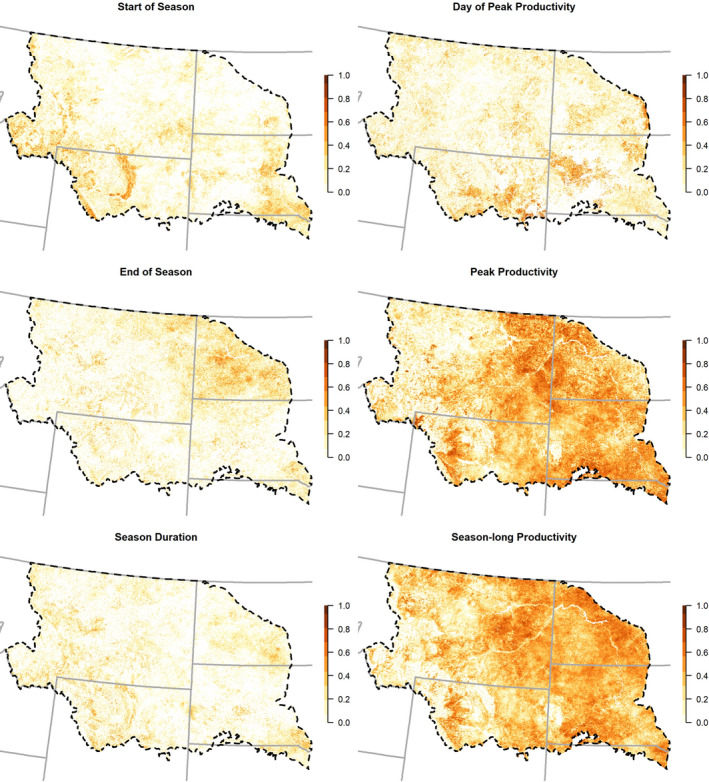
*R*^2^ values for multiple linear regression models of the change from mean annual temperature, annual precipitation, and time for phenological measures in the Northwestern Plains. The study area is depicted with a dashed black line and U.S. states with gray solid lines

**FIGURE A6:**
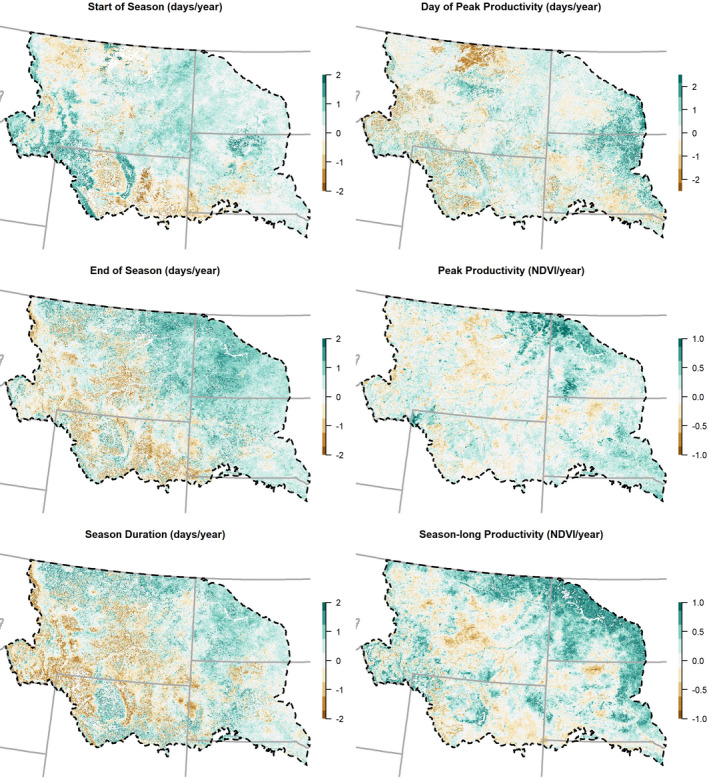
Slopes for change through time for phenological measures calculated from the AVHRR satellite record in the Northwestern Plains after accounting for annual precipitation and mean annual temperature. The study area is depicted with a dashed black line and U.S. states with gray solid lines. Note different axes for each plot

**FIGURE A7:**
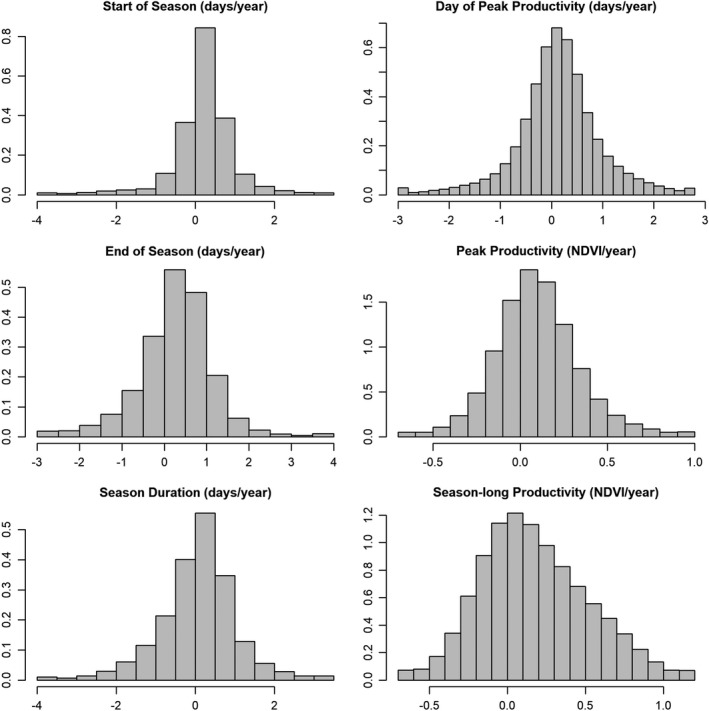
Histograms for the change over time for phenological measures calculated from the AVHRR satellite record for the Northwestern Plains after accounting for annual precipitation and mean annual temperature. Note different axes for each plot

**FIGURE A8:**
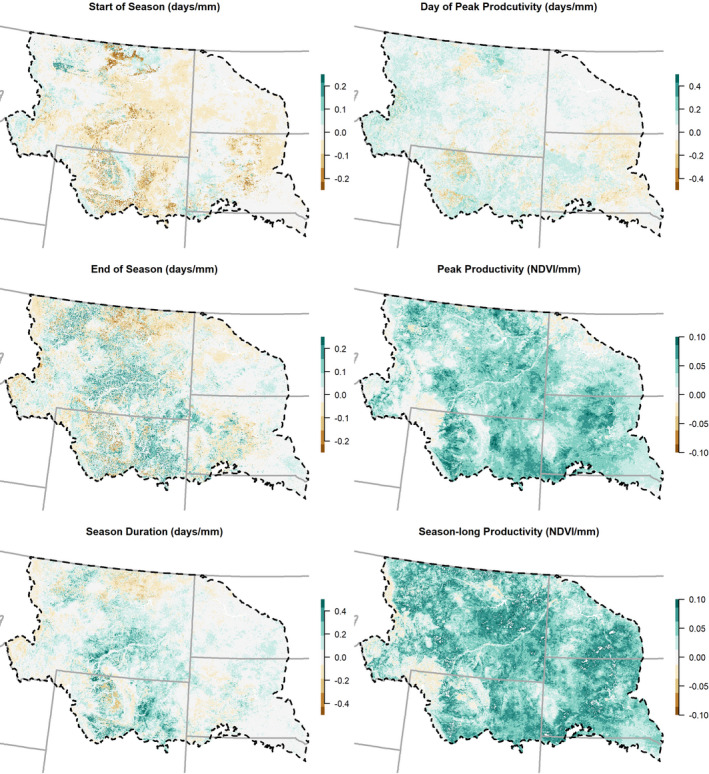
Slopes for the effect of annual precipitation on phenological measures calculated from the AVHRR satellite record in the Northwestern Plains after accounting for mean annual temperature and time. The study area is depicted with a dashed black line and U.S. states with gray solid lines. Note different axes for each plot

**FIGURE A9:**
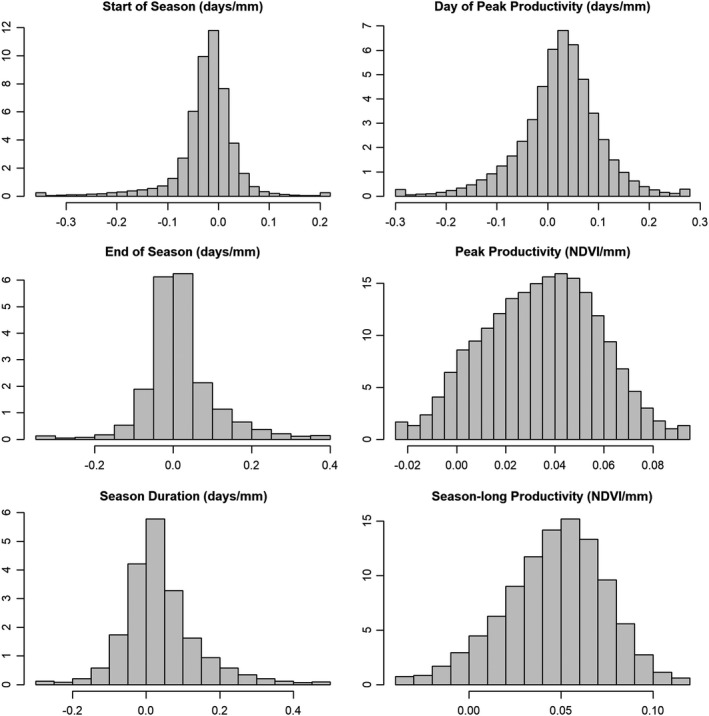
Histograms of the change from annual precipitation for phenological measures calculated from the AVHRR satellite record for Northwestern Plains communities after accounting for time and mean annual temperature. Note different axes for each plot

**FIGURE A10:**
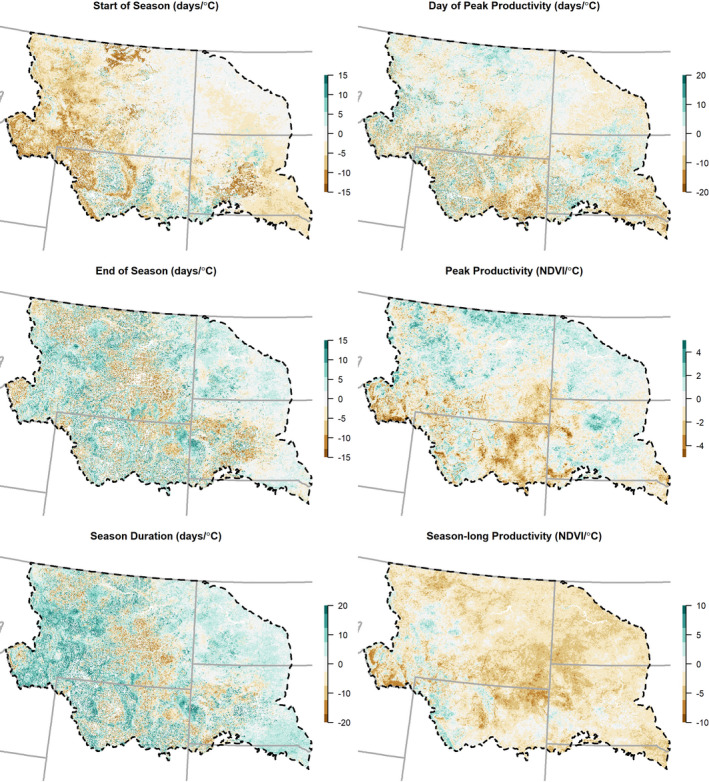
Slopes for the effect of mean annual temperature for phenological measures calculated from the AVHRR satellite record in the Northwestern Plains after accounting for annual precipitation and remaining time‐based trend. The study area is depicted with a dashed black line and U.S. states with gray solid lines. Note different axes for each plot

**FIGURE A11:**
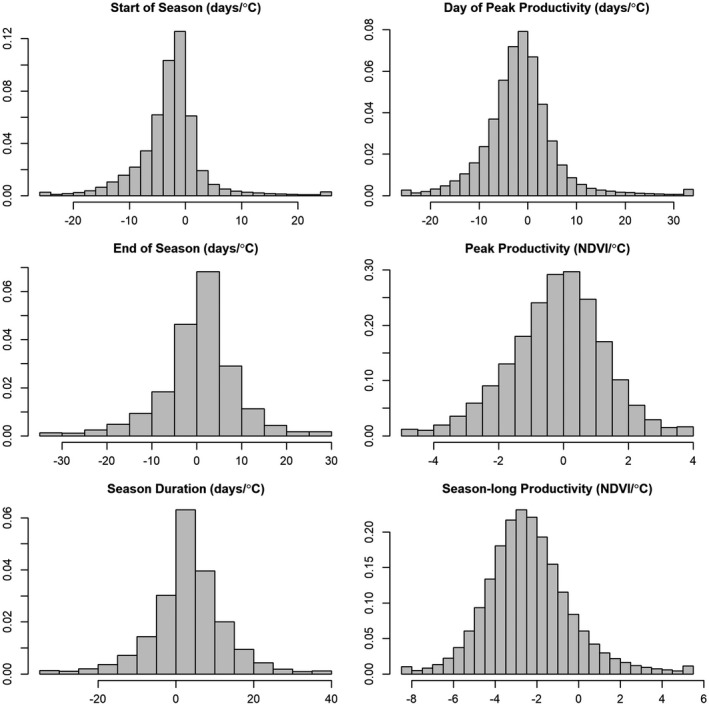
Histograms of the change due to annual mean temperature on phenological calculated from the AVHRR satellite record measures for Northwestern Plains communities after accounting for time and annual precipitation. Note different axes for each plot

Coefficients from our multiple regression analyses of phenology (Equation 1) varied across space, between each phenology measure, and across community types. Annual values of precipitation and temperature (Figure A1) in combination with time performed well in accounting for variation in peak productivity and season‐long productivity but had limited explanatory power for date‐based measures (Figure A5). After accounting for annual climate, many areas maintained strong associations with time (a surrogate for other potential effects not covered by precipitation and temperature) and in similar areas as those with significant trends in the interannual trend analyses (Figures A6 and A7). In general, peak productivity and season‐long productivity increased as annual precipitation increased (Figures 4, 5, A8, and A9). However, higher mean annual temperature tended to increase peak productivity yet decrease season‐long productivity (Figures 4, 5, A10, and A11). Higher mean annual temperatures were predominantly associated with earlier start‐of‐season dates, but changes to end of season and length of season were much more mixed between earlier/shorter and later/longer (Figures A10 and A11).

**FIGURE A12:**
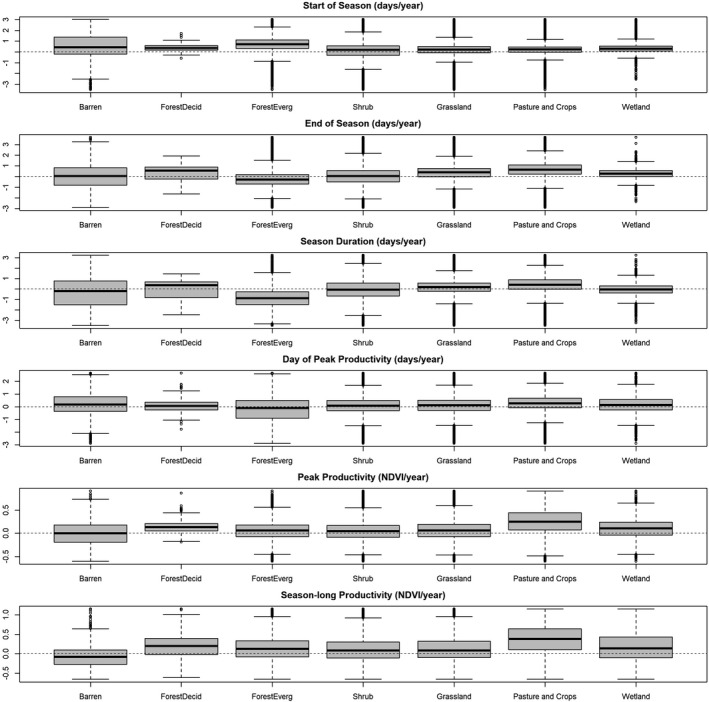
Boxplots for the change over time in phenological measures calculated from the AVHRR satellite record for major Northwestern Plains vegetation types (ForestDecid = Deciduous Forest, ForestEverg = Evergreen Forest, see text for description of each land‐cover class) after accounting for annual precipitation and mean annual temperature. Note different axes for each plot

**FIGURE A13:**
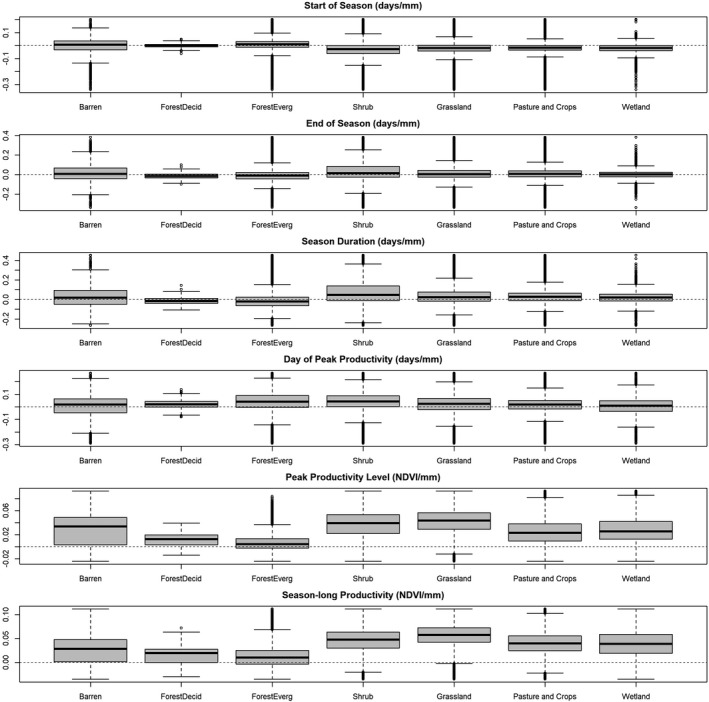
Boxplots for the change from annual precipitation on phenological measures calculated from the AVHRR satellite record for major Northwestern Plains vegetation types (ForestDecid = Deciduous Forest, ForestEverg = Evergreen Forest, see text for description of each land‐cover class) after accounting for time and mean annual temperature. Note different axes for each plot

**FIGURE A14:**
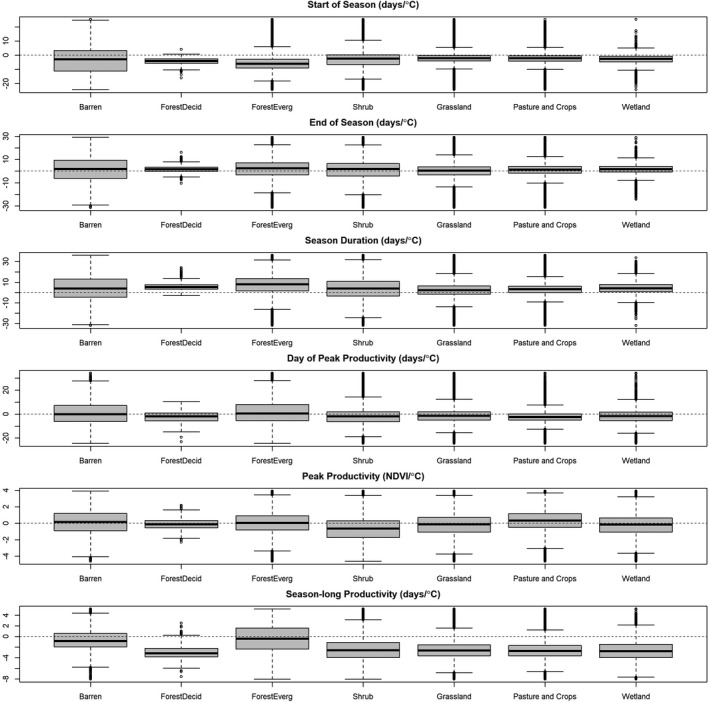
Boxplots for the change from mean annual temperature on phenological measures calculated from the AVHRR satellite record for major Northwestern Plains vegetation types (ForestDecid = Deciduous Forest, ForestEverg = Evergreen Forest, see text for description of each land‐cover class) after accounting for time and annual precipitation. Note different axes for each plot

The associations of phenological measures differed between community type (Figures A12, A13, A14). For example, evergreen forests tend to move in opposite directions as other community types with respect to time, precipitation, and temperature. Effect sizes, such as increased peak and season‐long productivity with time or increased precipitation, are larger in general for the pasture/crop areas and barren, shrub, and grassland communities, respectively. Season‐long productivity was more strongly associated with annual precipitation for all communities, whereas the strength of the associations of mean annual temperature and time varied between communities (Figures 4, 5, A12‐A14).

**FIGURE A15:**
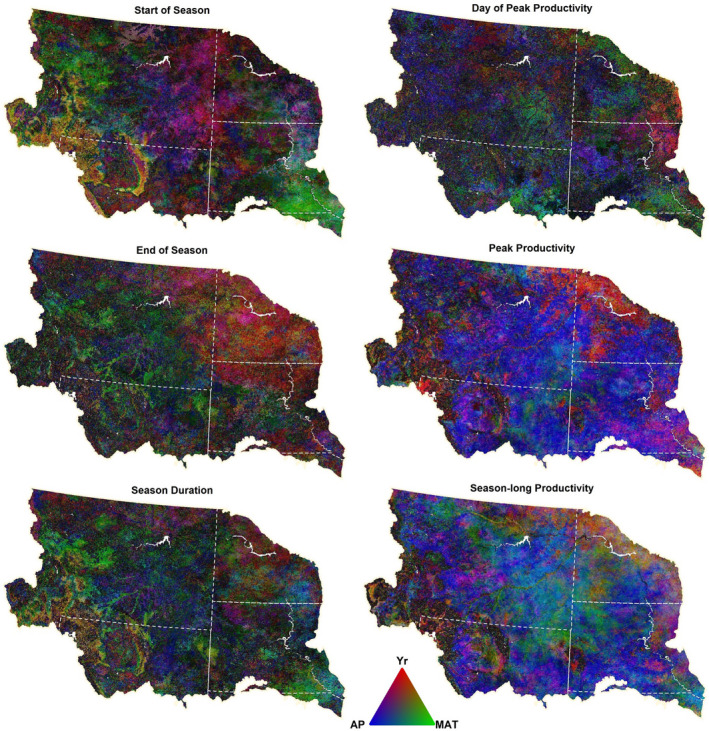
Combined partial correlations for time (Yr), annual precipitation (AP), and mean annual temperature (MAT) in phenological measures for the Northwestern Plains. Color represents the relative correlation of each factor in explaining the measures between 1989 and 2014. Brightness of the color is relative to the absolute value of the combined correlations where darker colors are smaller and brighter colors larger combined partial correlations. Dashed lines are U.S. state boundaries

In analyses investigating the relative contributions of all predictors through a partial correlation analysis, spatial patterns emerged for each of the six phenology measures (Figure A15). In the northeastern portion of the study area, time—a surrogate for multiple possible factors—was strongly associated with end‐of‐season dates, while areas farther west tended to have stronger associations with temperature (Figure A15). Temperature often had the largest partial correlation with date‐based phenological measures. However, for productivity‐based measures, precipitation had larger correlations with peak productivity, except in agricultural areas to the NE and forested areas in the SW (Figure 4). For season‐long productivity, temperature often in combination with annual precipitation had the larger partial correlations for most of the study area, with the same patterns for time as were seen for peak productivity (Figure 5). Within land‐cover types, the most prominent shifts in correlations due to precipitation for peak productivity versus precipitation and temperature for season‐long productivity occurred in the shrub, pasture/crop, and wetland communities (Figures 4 and 5).

## 4 DISCUSSION

We identified three main findings from this research: (1) Over our study period, we found that only a small proportion of our study area had significant temporal trends in any of our six phenological measures; (2) annual climatic variables had robust explanatory power for productivity‐based phenological measures; and (3) there was substantial spatial variability within vegetation groups and variability in how strongly vegetation phenological groups varied through time and responded to potential drivers. Collectively, these results highlight that phenological patterns depend on ecological context and also illustrate hierarchical relationships (regionally and across local communities). In addition, monitoring and assessment of ecosystems should incorporate differences between and within communities and acknowledge that vegetation responses to changes in climate may shift over time (e.g., Li et al., 2019; Yuan et al., 2019). These results are useful for management applications as they set the stage for efforts such as scenario planning. Some predictions of phenological changes through time, like potential future changes to productivity, may be identified using annual weather variables. Furthermore, analyses utilizing phenology such as models of ecosystem‐wide carbon uptake or responses of wildlife to climate change and variability should consider multiple phenological measures and context dependencies (Donnelly et al., 2018; Rehnus et al., 2020; Richardson et al., 2012). We discuss these three main findings in more detail in the following paragraphs.

First, assessment of interannual trends during 1989–2014 identified limited portions of the study area as having statistically significant changes in any of the six phenological measures using Mann–Kendall tests on Sen's slope calculations. In addition, for each of these significant pixel‐scale trends, there were differences in the magnitude and direction of change between vegetation types and across space. The drivers of phenological measures varied across the study area and communities, illustrating time trends alone in individual phenology‐based response variables did not reveal the complexity and trade‐offs between phenological measures in time and space (Butterfield et al., 2020; Wang et al., 2011; Wu et al., 2020).

Second, annual precipitation and temperature had strong explanatory power for productivity‐related phenological measures but tended to predict date‐based measures poorly. By including relatively simple explanatory variables, we were able to identify changes through time and across communities and to identify divergent influences of temperature on peak versus season‐long productivity. Therefore, the definition of greening and implications of both measures should be clearly understood and incorporated in phenological studies (Gao et al., 2020). Furthermore, illustrating the consequences of greening and/or browning under contemporary or future scenarios should include assessment of their patterns across varied plant communities and assess the trade‐offs/exchanges of higher spring/peak productivity for lower season‐long production (Butterfield et al., 2020; Hu et al., 2010; Wang et al., 2011).

Third, patterns of phenological measures and their relationship to climate varied within and across community types. Community responses to a changing climate can depend on context, such as latitude and elevation (Cowles et al., 2018; Reed et al., 2019). In some cases, disturbance and management decoupled phenology from climate and led to trends in phenology that could be mistakenly attributed when only considering interannual trends. Furthermore, additional drivers can also impact vegetation phenology via discrete events, such as fertilization, management, shifting community composition (including invasive species), or disturbance (Gu et al., 2003; Hao et al., 2019; Piao et al., 2019; Turner, 2010; Zhang, Liu, et al., 2019), all of which complicate temperature‐ and precipitation‐based explanations for phenological phenomena. Accordingly, specific drivers or surrogates such as time should be quantitatively included along with climate.

### 4.1 The role of climate in phenological variability

**FIGURE 6:**
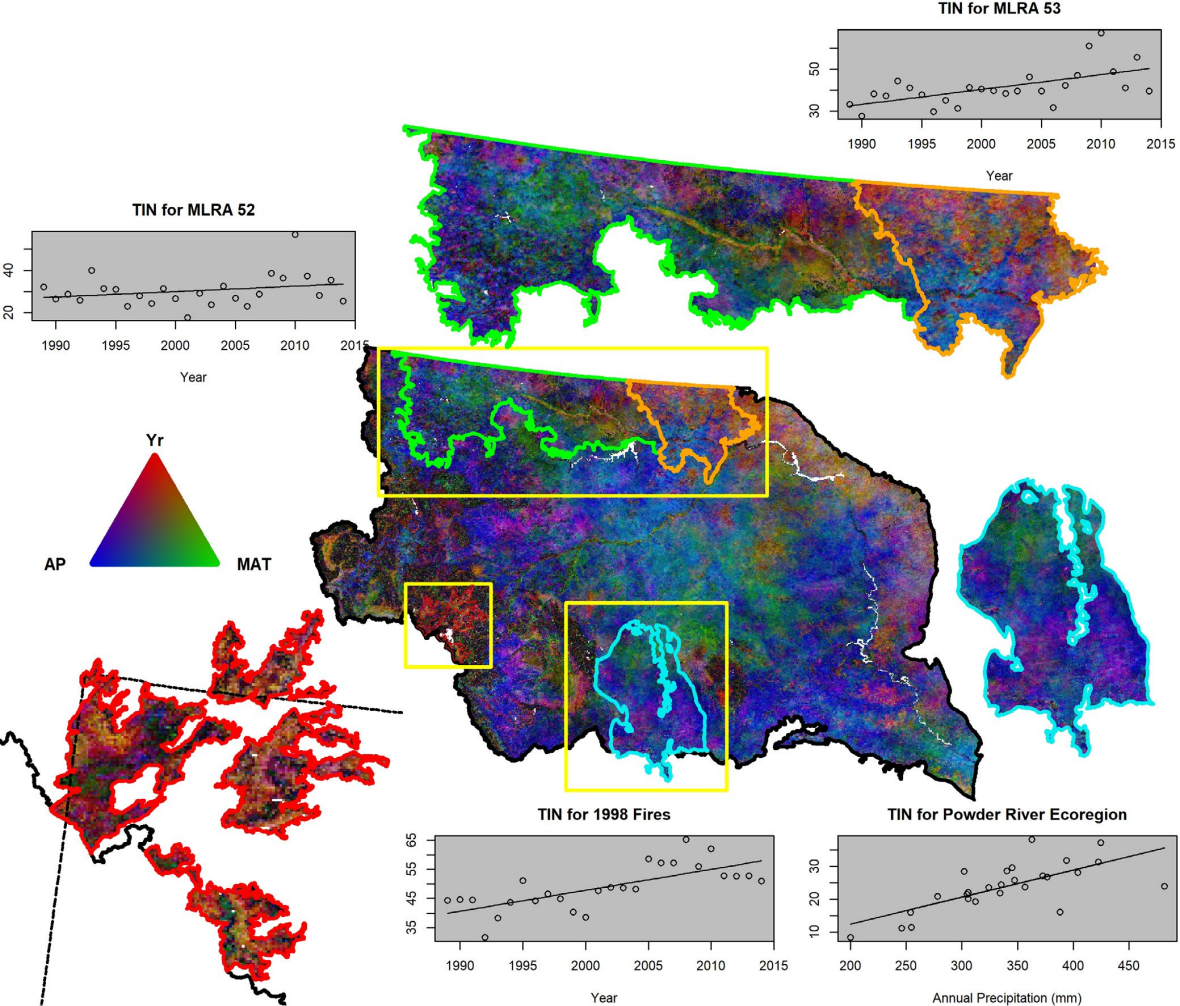
Display of partial correlation analysis of season‐long vegetation productivity containing examples of associations dominated by time, a surrogate for multiple potential factors influencing phenology, and by climate. Productivity is measured by AVHRR time‐integrated NDVI (TIN) from 1989 to 2014. The top breakout map and plots illustrate differences in agricultural practices between Major Land Resource Area (MRLA, https://www.nrcs.usda.gov/wps/portal/nrcs/main/soils/survey/geo/) 53 (orange boundary) where summer fallow has been decreasing and MLRA 52 (green boundary) where acreages in fallow are stable. The bottom left map and plot show recovery in Yellowstone National Park from disturbance (1988 fires, red boundaries, (Welty & Jeffries, 2000)) as productivity increased through time. The bottom right map and plot display how productivity increases with annual precipitation in the Powder River Basin Level 4 Ecoregion (light blue boundary, https://www.epa.gov/eco‐research/ecoregions). Yellow boxes in the central figure represent enlarged areas on the periphery. Scatter plots show TIN against time or yearly precipitation totals with a line derived from a simple linear regression. See Figure 5 for description of how colors represent the relative weighting of slopes from a partial correlation analysis of climate and time variables against TIN and the text for more details on each example

Increasing precipitation had a clear association with increased productivity across large areas of the NWP (Figure 5), such as in low elevation, dry, warm areas like the Powder River basin (Figure 6). We found that grasslands and shrublands had larger positive responses in both peak and season‐long productivity to precipitation relative to other communities (Figure A13). Precipitation is a strong driver of grassland productivity in the NWP (Petrie et al., 2016; Yang et al., 1998), and in other similar biomes, globally (Meng et al., 2020; Wu et al., 2020). Furthermore, responses by a community to water limitations, and therefore productivity, vary between communities (Maurer et al., 2020; Ponce‐Campos et al., 2013; Webb et al., 1978) and even between grassland types (Konings et al., 2017; Yang et al., 1998). Conversely increasing temperature mostly had a divergent association, increasing peak productivity but lowering season‐long productivity. Implications of warming temperatures on productivity can vary between vegetation functional groups (Livensperger et al., 2016), have cascading ecosystem effects (Beard et al., 2019; Rehnus et al., 2020; Renner & Zohner, 2018), and include consequences to human society (Bezerra et al., 2019; Stevenson et al., 2015).

Overall, both precipitation and temperature were needed to fully explain phenological measures (e.g., Gao et al., 2020). Spatial and ecological differences in limiting factors—such as temperature restrictions on the length of the season in water‐sufficient areas versus water‐limited systems with ample growing days—lead to spatial variation (Chen et al., 2019; Cowles et al., 2018; Piao et al., 2019; Reed et al., 2019; Yang et al., 2021). The precipitation and temperature controls on productivity are stronger in arid and semi‐arid regions such as much of the NWP (see Figure A1), compared to more mesic areas (Maurer et al., 2020). There are important trade‐offs that may come from shifts in the growing season (Figure 2; see also Fu et al., 2017; Butterfield et al., 2020). Specifically, whereas warm, wet springs may increase early‐season productivity and peak productivity, this may be offset by lower soil‐water recharge and decreased production during dry, hot summers (Butterfield et al., 2020; Hu et al., 2010; Lian et al., 2020; Wang et al., 2011). Conversely, a later start of spring from delayed snow melt can lead to increased, sustained, summer productivity (Hu et al., 2010; Potter, 2020). These relationships and potential trade‐offs may impose conflicting selection pressures on other members of the ecosystem due to asynchronous changes between start of season and peak versus season‐long productivity.

Further investigations of the role of vapor pressure deficit (VPD), or other metrics of atmospheric moisture and water balance, may help explain seasonal vegetative processes, beyond those informed by temperature and precipitation. In North American grasslands, VPD during July and August was a strong predictor of productivity (Konings et al., 2017). Higher VPD leads to decreased gross primary productivity across global ecosystems (Zhang, Ficklin, et al., 2019), and VPD even was a strong predictor in small mammal distribution models (Johnston et al., 2019; Smith et al., 2019). In our study area, northeastern Montana and much of North and South Dakota showed decreasing VPD between 1979 and 2013 in the spring and summer, while VPD in central Montana and Wyoming increased (Bromley et al., 2020; Ficklin & Novick, 2017). Although VPD is a promising explanatory variable for vegetation responses to climate, it is unclear how to best aggregate VPD across time to account for its nonlinear impacts on productivity (Lasslop et al., 2010).

### 4.2 Nonclimate associations

We included time in our models as a surrogate for multiple potential linear factors other than temperature and precipitation that can influence phenological measures, and in the NWP time strongly governed response in specific areas (Figures 4, 5, 6). Fire and insect disturbances are common in forests of the NWP and may have affected productivity (Pfeifer et al., 2011; Turner, 2010). For example, the Greater Yellowstone Ecosystem experienced stand‐replacing fires across large areas in 1988 that were followed by reestablishment of forest communities such as those dominate by lodgepole pine (*Pinus contorta*) (Turner, 2010). We found stronger associations between time and season‐long productivity within fire perimeters compared to outside, illustrating postdisturbance succession such as from patchy pioneer plant species to dense early‐stage evergreen forests (Figure 6).

Likewise, shifts in agricultural practices such as decreased summer fallow (Lubowski et al., 2006; Vick et al., 2016), increased cropping efficiency (Ray et al., 2012), changes to crop type (e.g., different sowing dates) (Zhang, Liu, et al., 2019), and field abandonment/human movement (Hao et al., 2019; Kolecka, 2021; Li et al., 2017) can impact phenological measures. However, these changes are not spatially uniform. For example, in northeastern Montana (Figure 6) Major Land Resource Area (MLRA) 52 has had stable amounts of fallow acres, but to the east in MLRA 53 fallow acreages have declined in favor of increased cover crops (Long et al., 2014; Vick et al., 2016). In areas of declining fallow, increased vegetation leads to greater photosynthesis and productivity in the growing season, while areas without declines in fallow would exhibit more stable amounts of vegetation and therefore are more strongly controlled by precipitation. Importantly, planting and harvesting decisions can play a large role in the phenology of agroecosystems; such decisions exhibit finer level changes than those typically identified through land‐cover classifications. Overall, portions of the study area that have strong time‐based controls over season‐long productivity often have logical explanations based on known disturbances and management practices, which tend to decouple phenology from climate.

### 4.3 Opportunities for application and addition study

We used annual values for temperature and precipitation to test the improvement from interannual trends with only a small increase to the dimensionality of our models. Although this approach had strong explanatory power for productivity measures, it was more limited for date‐based phenological measures. Phenology is responsive to seasonal conditions prior to and during the growing season that may diverge from annual patterns (e.g., Bianchi et al., 2019; Fu et al., 2017; Ren et al., 2020), and although there is often strong correlation between annual and seasonal values, climate trends and species response to climate may not vary homogeneously (Bromley et al., 2020). Temperature was the clearer control on season duration: Warmer temperatures can advance spring (Allstadt et al., 2015; Fu et al., 2017; Ren et al., 2020) and extend the growing season through longer snow‐free periods and later freeze events (Potter, 2020). Later spring starts and spatial differences in the direction of spring movement are not unprecedented in the Northern Hemisphere (e.g., Li et al., 2019; Ren et al., 2020). For example, spring is trending earlier in western and central Europe but later in eastern Europe, attributable to differing weather patterns (Ahas et al., 2002). Shifts to later end‐of‐season dates (as found in much of our study area) are found in central Asia and China, and movement toward earlier end‐of‐season dates is found in more southern areas of the Great Plains, western and central Asia, and parts of Mongolia (Ren et al., 2020). Overall, more complex models addressing seasonal time periods (Ren et al., 2020), additive growing degree days (de Beurs & Henebry, 2007), and/or ecological memory (Liu et al., 2019) have promise for date‐based phenological measures.

In our study, we identified that trends toward higher peak productivity did not always translate to higher season‐long production. Drivers of peak and season‐long productivity can differ in general and across vegetation communities (Fu et al., 2017, 2019; Xia et al., 2015). Mixed responses of greening versus browning in grassland, shrub, steppe, and forested areas are also found in regions of Patagonia (Bianchi et al., 2019) and across vegetation communities in Mongolia (Meng et al., 2020). We demonstrated that greening and browning studies should consider peak and season‐long productivity differently, given that their relationships to climate drivers differed markedly. Earlier or higher growth and/or longer growing‐season peaks can be offset by limited production in a dry summer (Livensperger et al., 2016; e.g., Hu et al., 2010). These shifts, trade‐offs, and asynchronies in vegetation phenology have feedbacks and consequences to ecological communities, including consumer–resource dynamics, species' behaviors, and other ecosystem processes (Beard et al., 2019; Morisette et al., 2009). Understanding the implications of future climate change is important for predicting potential ecological and social‐ecological impacts (e.g., Allstadt et al., 2015; Beard et al., 2019; Beever et al., 2019; Epstein et al., 2021; Hufkens et al., 2016). A collective understanding and consideration of multiple phenological measures and their drivers is important for effective management actions and continued development of a multiscale ecological perspective for management (Bezerra et al., 2019; Morisette et al., 2009).

## CONFLICT OF INTEREST

None declared.

## AUTHOR CONTRIBUTIONS

**David J. A. Wood:** Conceptualization (equal); data curation (equal); formal analysis (lead); investigation (lead); methodology (lead); resources (equal); writing‐original draft (lead); writing‐review & editing (equal). **Scott Powell:** Conceptualization (equal); investigation (supporting); methodology (equal); supervision (equal); writing‐original draft (supporting); writing‐review & editing (equal). **Paul C. Stoy:** Conceptualization (equal); investigation (supporting); methodology (equal); supervision (equal); writing‐original draft (supporting); writing‐review & editing (equal). **Lindsey L. Thurman:** Data curation (equal); formal analysis (supporting); methodology (equal); writing‐review & editing (equal). **Erik A. Beever:** Data curation (equal); methodology (supporting); resources (equal); writing‐review & editing (equal).

## Data Availability

Data are accessible from public repositories with details on processing in the manuscript. Land surface phenology data and fire perimeter data are available from the U.S. Geological Survey at https://doi.org/10.5066/F7PC30G1 and https://doi.org/10.5066/P9Z2VVRT. Land‐cover and land‐use data are available from the Multi‐Resource Land Characteristics Consortium at https://doi.org/10.5066/P937PN4Z. Climate data are available from the PRISM Climate Group at Oregon State University (https://prism.oregonstate.edu/). However, free data are only available at a 4 km resolution, and the 800 m gridded data used herein can be procured from the PRISM group. Ecoregional boundaries were downloaded from the Environmental Protection Agency (https://catalog.data.gov/dataset/u‐s‐level‐iii‐and‐iv‐ecoregions‐u‐s‐epa) and Major Land Resource Area boundaries from the National Resource Conservation Service (https://catalog.data.gov/dataset/major‐land‐resource‐areas‐mlra).

## APPENDIX A

### Additional spatiotemporal and community patterns and findings

These figures and tables provide additional information on temperature and precipitation patterns, potential contributions of land‐cover change, differences between communities, and distributions of slopes from the multiple linear regression analyses. In addition, results for all phenological measures are included from the partial correlation analyses showing the spatial differences between predictors.

**TABLE A1:** Distributions of significant trends (*p* < .05, Mann–Kendall test) across community types with direction of Sen's Slopes, for 1989–2014 for six phenological measures based on the AVHRR satellite record

	Vegetation community type							
	Barren	Deciduous forest	Evergreen forest	Shrub	Grassland	Pasture/Crop	Wetlands	Total
Start of season								
Significant	12%	11%	13%	14%	8%	6%	9%	9%
Increasing (later)	88%	100%	95%	52%	70%	75%	94%	70%
Decreasing (earlier)	12%	0%	5%	48%	30%	25%	6%	30%
End of season								
Significant	6%	28%	6%	9%	13%	18%	10%	13%
Increasing (later)	43%	43%	17%	91%	97%	98%	97%	92%
Decreasing (earlier)	57%	57%	83%	9%	3%	2%	3%	8%
Season duration								
Significant	6%	6%	7%	5%	2%	4%	2%	4%
Increasing (longer)	30%	37%	4%	73%	90%	94%	49%	68%
Decreasing (shorter)	70%	63%	96%	27%	10%	6%	51%	32%
Day of max NDVI								
Significant	2%	2%	5%	3%	4%	13%	5%	6%
Increasing (later)	53%	22%	18%	29%	49%	67%	52%	53%
Decreasing (earlier)	48%	78%	82%	71%	51%	33%	48%	47%
Maximum NDVI								
Significant	21%	42%	24%	17%	28%	49%	26%	29%
Increasing	49%	100%	73%	92%	98%	99%	96%	95%
Decreasing	51%	0%	27%	8%	2%	1%	4%	5%
Time‐integrated NDVI								
Significant	4%	20%	10%	11%	21%	47%	20%	23%
Increasing	27%	99%	87%	86%	96%	98%	88%	96%
Decreasing	73%	1%	13%	14%	4%	2%	12%	4%

Note

1—Increasing and decreasing values are calculated from only significant pixels, with total trends being the number of significant pixels divided by the total number pixels of the community type. For each community type, the percentage increasing (decreasing) is the number of significant pixels with a positive (negative) slope divided by the total number of significant pixels. Therefore, increasing slopes correspond to transition dates later in the year, longer seasons, or higher productivity as applicable to each phenology measure.
